# Impact of food processing on the allergenic properties of amylase trypsin inhibitors from wheat

**DOI:** 10.3389/falgy.2023.1228353

**Published:** 2023-11-22

**Authors:** Peter L. Weegels, Antoine H. P. America

**Affiliations:** ^1^ Laboratory of Food Chemistry, Wageningen University and Research, Wageningen, Netherlands; ^2^ Plant Breeding, Wageningen University and Research, Wageningen, Netherlands

**Keywords:** ATI, food processing, extractability, wheat allergy, non-coeliac wheat sensitivity

## Abstract

Amylase trypsin inhibitors (ATIs) play an important role in wheat allergies and potentially in non-coeliac wheat sensitivity. Food processing could be important to mitigate the pathogenic properties of ATIs, e.g., by denaturation, glycation, enzymatic hydrolysis, cross-linking, and oxidation and reduction. These modifications also impact the solubility and extractability. The complex solubility behaviour of ATI isoforms (water and salt soluble, but also chloroform–methanol soluble, solubility depending on the redox state) becomes even more complex upon processing due to denaturation and (bio)chemical modifications. This significantly hinders the feasibility of quantitative extraction. Moreover, changes in biofunctionality may occur during the process of extraction, and the changes in ATI due to food processing will be more difficult to assess. Heat treatment decreases the extractability of ATIs with water, NaCl, and other buffer extracts, and binding of IgE from wheat-allergic persons to ATIs as observed with Western blotting is decreased or absent. IgE binding is reduced with the total extract in chaotropic and reducing agents. However, it can be increased when the proteins are hydrolyzed by proteases. Fermentation involving certain species of *Fructolactobacilli* (FLB), followed by baking, decreases the amount of ATIs and IgE binding to ATIs. In yeast-fermented bread, the amount of ATIs decreased in a similar manner, but IgE binding was more prominent, indicating that there was a modification of ATIs that affected the epitope recognition. When isolated ATIs are ingested with high ATI degrading FLB, the immune response in mice is less elevated *in vivo*, when compared with ATI without high ATI degrading FLB. The pathogenic effects on the skin of dogs and one wheat-allergic child are also decreased when soluble proteins or isolated ATIs are reduced with the thioredoxin/thioredoxin reductase NADPH system. Glycation on the other hand has been shown to potentiate the allergenic properties of ATIs as evidenced by the large increase in IgE binding. The impact of food processing on the pathogenic properties of ATIs is hardly studied *in vivo* in humans. There seem to be opportunities to mitigate the pathogenic properties *in vitro*, but potentiation of pathogenic properties is also frequently observed. This requires a deeper understanding on the impact of food processing on the pathogenicity of ATIs.

## 1. Introduction

Wheat is the most important staple food consumed in the Western world (providing 20%–25% of the daily energy intake). It significantly contributes to our protein, vitamin, mineral, and fibre intake and is the most abundant source of plant protein (1). Wheat products, on the other hand, are a source of concern for some consumers. In addition to coeliac disease (CD) and wheat allergy, intolerances such as non-coeliac wheat sensitivity (NCWS) and irritable bowel syndrome (IBS) have also been related to wheat consumption. NCWS and IBS affect 5%–30% of the population, resulting in a lower quality of life, reduced productivity, increased healthcare costs, and overall economic burden. In the EU, the estimated yearly direct and indirect costs associated with NCWS and IBS range from €90 to €560 billion [calculated from (2)]. It also deprives these patients from their main staple food, reduces the intake of important nutrients, and forces them to consume more expensive alternatives. In many cases, gluten proteins (gliadins and glutenins) have been considered to be responsible for inducing the negative responses. However, recent *in vitro* studies have indicated that amylase trypsin inhibitors (ATIs) may have a significant role in the development of symptoms, not only in wheat-induced allergies and CD, but also in NCWS and IBS (3–5). An important route to mitigate their potentially pathogenic effects is by food processing (6). Understanding the role of ATI in cereal-based food processing and food digestion, and mitigation of the negative effects, is therefore of prime importance for cereal-based food safety, security, and sustainability. The impact of food processing on ATIs and their potential pathological effects will be reviewed. In addition to the opportunities, the limitations will be discussed, encompassing both the methodological and processing-related aspects.

## 2. Presence and content of ATI in wheat: extractability is key

ATIs are part of the albumin and globulin fraction of wheat. Unlike storage proteins, ATIs exhibit minimal variation in content across different wheat varieties and agronomic conditions, such as fertilization (7, 8). Several methods for isolating wheat proteins have been proposed to establish their content. A century ago, Osborne proposed a variety of extraction procedures, with the most frequently employed method yielding protein fractions extracted by the use of a saline solution, followed by a further water extraction to obtain globulins and albumins. Gliadins were extracted using 70% ethanol, while the remaining protein fractions were referred to as glutenins (9). The albumin/globulin fractions contain most of the ATIs. One of the primary challenges in extracting ATIs is the need to achieve a quantitative extraction and obtain fractions that are as pure as possible. More pure fractions as determined by SDS-PAGE, RP–HPLC, and LC–MS/MS could be obtained by other procedures, e.g., ammonium urea cetyltrimethyl bromide extraction followed by ammonium sulphate precipitation (10, 11), or by SDS/DTT extraction and separation by RP–HPLC (12, 13). The procedures using chaotropic and reducing agents extract most of the ATIs from flour (12, 13) and baked bread (13).

The diversity of the ATI isoforms (11, 14–17) is extensive, with a reported number of up to 90 isoforms (14), while only approximately 20 isoforms have been studied in detail and are classified according to their mono, di, or tetrameric state [(18) Table 1]. The isoforms have a substantial homology, and their identification and quantitation by LC–MS/MS should be regarded with caution. In various species of wheat, various identical ATI peptides have been found, but the ratios between those peptides vary depending on the species (19). This finding indicates that there are multiple isoforms, including those that are not yet documented in protein sequence databases, which share several peptides. Consequently, relying on a limited number of peptides to identify complete protein species should be regarded with caution. As not all isoforms are reported in the protein sequence databases and the annotation of peptides to the isoforms is quite complex, if not impossible, it makes also identification and quantitation of isoforms difficult.

**Table 1:** Classification of common ATI isoforms and selected properties.

Aggregation state	Protein subunit	Widely used names	Functionality	Predominant solubility
Monomeric	WMAI-1	0.28	Amylase inhibition	Water, saline
Homodimer	WDAI-1	0.53	Amylase inhibition	Water, saline
	WDAI-2	0.19		
Tetramer 1st subunit	WTAI-CM1	CM1	Amylase inhibition, CM16* strong IgE binding	Water, saline, chloroform–methanol
	WTAI-CM2	CM2		
2nd subunit	WTAI-CM16	CM16		
	WTAI-CM16 glycosylated form	CM16*		
	WTAI-CM17	CM17		
3rd subunit (2 copies)	WTAI-CM3	CM3		
Monomeric	CMx1/2/3	Trypsin inhibitors	Trypsin inhibition	

The table is based on (14) and (18) with adaptations.

* symbol notifies glycation in (27).

The isoforms not only vary in their amino acid sequence and secondary structure, but also in their tertiary and quaternary structure (mono-, di-, or tetramer) and posttranslational modification (phosphorylation, glycation). The isoforms are likely to vary in their enzyme inhibitory activity, allergenicity, extractability, and complexation behaviour.

ATI isoforms show anomalous extraction behaviour. Although water and saline extractable, some isoforms are soluble in chloroform–methanol mixtures (20), and ATIs can be found in relatively large quantities in the water-insoluble protein, known as gluten; in the 70% ethanol extracts of flour, the gliadin fraction; in the SDS extractable glutenin fraction; or the SDS unextractable protein, the glutenin macropolymer (12, 13). It was estimated (based on UV absorption measurement calibrated with Kjehldahl method) that the content could be as high as 1.5%–3.3% of *Triticum aestivum* flour (21, 22). Based on total solubilization, precipitation, and protein quantification using the Kjehldahl method, it could be calculated that the wheat kernel contains 0.6%–0.8% of ATIs (11). The LC–MS/MS quantifications (with peptide-targeted detection calibrated by isotope dilutions) have yielded estimations of much reduced quantities, approximately 0.4%–0.5% of wheat, spelt, durum, or emmer flour. Einkorn did not contain detectable amounts of ATIs (23).

Furthermore, the redox state of ATIs alters their extractability substantially, e.g., disulphide bond reduction of ATIs that are methanol extractable converts these ATIs to methanol unextractable (24). Therefore, it is likely that the extractability changes when the food matrix changes in redox potential, e.g., with the use of reducing or oxidizing agents either added as ingredients during processing or from microorganisms. It can also impact solubility and quantitation from 2D gel electrophoresis in which typically proteins are applied unreduced in one dimension and reduced in the other. Undesired extraction of ATIs and undesired differential extraction of the ATI isoforms from gels can occur, particularly after prolonged destaining using water or methanol washing as ATIs can be extractable in these media.

Denaturation of proteins results in an alteration of the three-dimensional structure and can be caused by heat, (bio)chemical reactions, high pressure, or high shear. Heat treatments that are typical during food processing can affect protein extractability in several ways:

• Heat treatment can change the conformation and ultimately can denature protein, making them unextractable.
• Cross-linking with other proteins can decrease extractability via disulphide bond formation or other oxidation reactions
• Lysinoalanine and lanthionine formation (25).
• Due to denaturation of proteins and gelatinization of starch, proteins can lose extractability due to physical entrapment. This was also demonstrated for ATIs (13).
• During heat treatment, proteins can react with glucose or other carbohydrates (26) according to the Maillard reaction, and indeed the ATI isoform CM16 can become glycated (27). The impact on extractability of this modification is not clear.
• Finally, during food processing, reducing and oxidizing agents are used to improve processing and transformation of foods. This can also alter the redox state of ATIs and thereby their extractability (24).

The reduced solubility of ATIs and hence the extractability from a processed food matrix will not only influence its quantitation. It also seriously hampers the assessment of the biofunctionality of ATIs, as many methods (enzyme inhibition, cell line studies, reactivity with antibodies) require a solubilized protein that is kept in its molecular state as it was *in situ* in the processed food. In many studies, only chaotropic and reducing agents could be used to solubilize proteins, and one can question the *in situ* biofunctionality of these proteins once extracted from the food. Thus, a large portion of ATIs can only be extracted from baked bread when SDS/DTT is used (13) or when the unextractable ATIs are extracted after sonication (13), which is able to excessively break peptide bonds (28).

A reduction in enzyme inhibition after processing, e.g., by a change in protein conformation, does not automatically lead to a loss of allergenicity or pathogenicity. On the contrary, hidden allergenic epitopes can become exposed upon partial hydrolysis or after disulphide bond reduction. Heat treatment of gliadins reduced the recognition by IgE, but subsequent pepsin hydrolysis partly recovered recognition (19). Similar studies with ATIs have not been encountered in the literature search. Glycation can lead to a major increase in allergenicity as shown by the increase in the binding of IgE from Baker's asthma patients upon glycation of CM16 (27).

Finally, a limited degree of enzymatic hydrolysis can reduce the quantity of intact proteins and generate fragments that cannot be detected with normal SDS-PAGE and Western blot; therefore, seemingly binding of IgE (from people with wheat allergies) is lost.

The issues mentioned in this section should be taken into account when reviewing the effect of food processing on ATI biofunctionality. It should also be realized that the impact of processing may vary substantially between the ATI isoforms. A limited number of publications were encountered that described the impact of processing on individual isoforms.

## 3. ATIs during food processing

Treatments to mitigate the negative effects of ATI have been studied in model systems:

• reduction of disulphide bridges [a.o., by treatment with thioredoxin (29–31)].
• proteolysis by pepsin [most other proteases are inhibited by ATI (21)].
• chemical modification (32).
• prolonged heating at high water activity (33–36), leaching of ATI during boiling (37, 38).
• microbial fermentations (39, 40).

When isolated ATI was heated, it retained to a large extent its inhibition of amylase and trypsin. The inhibitory activities were also not lost with the reducing agent β-mercaptoethanol. The inhibitory activity was only lost when β-mercaptoethanol was applied at high temperatures (41). When mice were fed a combination of isolated ATIs and *Fructolactobacilli* (FLB) that have high capacity to degrade ATIs, there was a significant reduction of 55% in the inflammation marker tumour necrosis factor-α (TNF-α). In addition, the increase of interleukin 6 (IL6) was approximately 50% lower compared with mice that were fed isolated ATIs without FLB (40).

Enzymatic oxidation by horseradish peroxidase has been shown to be effective in eliminating the enzyme inhibitory activity of ATI, by oxidizing one methionine and two tryptophan residues (32, 42). Inhibitor proteins from several sources (wheat alpha amylase inhibitors, soybean Bowman–Birk trypsin and Kunitz inhibitors, and corn kernel trypsin inhibitor) undergo reduction by thioredoxin in combination with DTT or NADP–thioredoxin reductase (NTR). It was speculated that this could regulate the enzyme inhibitory activity (43). It has been shown that thioredoxin treatment in combination with DTT or NTR of wheat proteins can mitigate the allergic properties in skin prick tests with dogs (31). With gliadins, glutenins, and globulins, the mitigation was complete below dosages of 1–3 ng in a highly sensitive dog. With albumins, the mitigation varied between 46% and 69% below this range (31). One case has been described in humans, in which skin wheal (urticaria) reactions markedly decreased and *in vitro* binding of IgE from a child with baker's asthma to wheat was reduced after the NTR treatment of ATIs (29).

Not all of these treatments can be used in cereal food processing or do not provide adequate mitigation of the negative effects.

In Table 2, an overview is given regarding the impact of various processing methods on the quantity and functionality of ATIs. As the extractability of ATIs may vary depending on the processing method, a difference is made between total extract (typically with SDS and reducing agents) and partial extract. Particularly in the latter case, changes in extractability can affect functionality. The albumin and globulin fractions (water and NaCl extracts) are found to contain a large amount of ATIs, but may not be quantitative after food processing procedures. Water and salt extracts also contain other components that can affect the functionality. Still, this review includes studies of the albumin/globulin fractions that do not quantify the ATIs, because they utilize SDS-PAGE or 2D electrophoresis in which the impact on the monomeric ATIs in the 14 kDa region can be observed. The impact of food processing on ATIs in these studies will be reviewed in qualitative terms. In the succeeding sections, the effect of food processing (fermentation, fermentation and baking, heating, general treatments) is discussed.

**Table 2:** Impact of wheat processing on the quantity and functionality of ATIs in total and partial extracts.

			Impact on ATI		
Processing treatment	Product	Extraction	Quantity (SDS-PAGE, ELISA, LC–MS/MS)	Wheat-allergic patient IgE binding (Western blot), *in vitro* cell line, SPT, enzyme inhibition	Reference
Fermentation total extract					
FLB fermentation	Wheat dough	SDS/DTT		IgE reduced	(44)
Yeast fermentation	Wheat dough	SDS/DTT		IgE present	(35)
FLB and yeast fermentation	Wheat dough	SDS/ME	Increase (FLB); equal (yeast)		(45)
FLB and yeast fermentation	Wheat dough	Ammonium bicarbonate ATI isolate		Decreased TNF-α, MCP-1 in FLB vs. yeast	(46)^a^
FLB, *Bacillus* spp., *Clostridium*, *Staphylococcus*	None	Isolation (>60% ATI)	0%–95% decrease depending on bacteria		(40)
Yeast fermentation and proteolysis	Wheat dough	SDS/ME		IgE decreased with pepsin and pancreatin hydrolyses, some ATIs were resistant	(37)
Fermentation partial extract					
Yeast and FLB fermentation	Wheat flour, dough	NaCl		IgE absent with FLB	(47)
FLB fermentation	Wheat dough	Tris-HCl	0%–98% reduced		(48)
FLB and Yeast fermentation	Wheat dough	NaCl	Decrease (FLB); equal (yeast)		(45)
FLB fermentation	Wheat dough	NaCl	41% reduction		(22)
FLB and yeast fermentation	Wheat dough	NaCl	Decreased tetrameric in FLB vs. yeast	22%–29% less amylase inhibition in FLB (yeast no results)	(46)
FLB fermentation	None	Isolation (>60% ATI)		*In vivo* mice: decreased TNF-α and 50% reduced increase IL6 with high ATI degrading FLB	(40)
FLB fermentation	Wheat dough	Chloroform–methanol	Reduction 22%–70% ELISA, 52%–85% RP–HPLC		(49)
Fermentation and baking; total extract					
Yeast fermentation, baking	Bread	SDS/DTT		IgE absent in crust and crumb	(35)
Yeast fermentation, baking, proteolysis	Bread	SDS/DTT		IgE increase when treated with pancreatin	(35)
FLB and yeast fermentation and baking	Dough and bread	SDS/DTT	Equal after proofing (FLB and yeast), 40%–70% reduced after baking (FLB and yeast); yeast equal FLB		(50)
Yeast and FLB fermentation, baking	Bread	SDS/ME	reduced in yeast and FLB bread	IgE reduced in yeast bread, absent in FLB bread	(47)
Fermentation and baking; partial extract					
FLB and yeast fermentation and baking	Bread	Tris-HCl/EDTA	Reduced (FLB and yeast), FLB more monomers, yeast more di/tetrameric		(51)^b^
FLB and yeast fermentation and baking	Dough and bread	Ammonium bicarbonate/Iodoacetamide	equal CM3 after proofing (FLB and yeast), 90%–95% reduced after baking (FLB and yeast); yeast less CM3 than FLB		(50)
Fermentation and baking	white, wholemeal, rye and mixed cereals dough and bread	Sodium acetate		Trypsin (TI)/Chymotrypsin (CI) inhibition dough < flour < bread. Wholemeal no TI; TI rye > wheat/mixed cereals > wholemeal; CI wheat > rye > wholemeal > mixed cereals	(52)
Heating, boiling, drying)					
Boiling	Wheat flour	SDS/DTT		IgE equal	Pastorello et al. 2007
Boiling	Durum flour	SDS/ME		IgE absent	(38)
Drying	Durum pasta	SDS/DTT	Decrease >110′C	IgE absent	(53)
Boiling	Durum pasta	NaCl	Decrease, ATIs in cooking water		(54)
Heating mixolab	Wheat dough	various	40% increase unextractable albumins/globulins upon mixing and heating; 5% increase extractable albumins/globulins upon mixing and heating		(13)^a^
Heating mixolab	Wheat dough	SDS/DTT	Absent in dough, 40% decrease 0.19 or 39%–332% increase 0.19, dimeric, CM3 and CM16		(13)
General; partial extracts and isolated ATI					
Baking, toasting, boiling	Bread, pasta, biscuits	NaCl	Reduced: boiling/toasting > baking alone > unboiled pasta and biscuits; indications for other epitopes after heat treatments	IgE absent in breads, pizza and boiled pasta, present in fresh, white and wholemeal pasta; SPT: flour vs. wholemeal tinbread equal, toasting wholemeal tinbread and boiling pasta: reduced	(34)^a^
Baking, boiling	Bread, pasta	Phosphate		Inhibition 80%–90% reduced in white and rye bread, 100% in wholemeal bread and 98% in spaghetti	Granum 1978
Baking, drying	Bread, pasta, cookies, cake	70% ethanol		No α-amylase inhibition in cake, cookies, crackers, muffin pretzel, cooked pasta, some types of breads, α-amylase inhibition in most types of breads (incl. flour as decoration), uncooked pasta, cous-cous	(55)
None	Wheat flour	NaCl (extractable), isopropanol/DTT (unextractable)		IgE none with unextractable: 0% (*n* = 20); partial extract 60% (*n* = 20) of patients	(56)
None	*Triticum aestivum* and *T. monococcum*	isolation		Increased TNF-α, IL8 in *T. aestivum* vs. *monococcum*	Iacomino et al. 2021
Thioredoxin	Salt-soluble proteins	NaCl		SPT human: abolishment of reaction	Matusmoto et al. 2007^a^
Thioredoxin	Salt-soluble proteins	NaCl		*In vivo* dog: reduction of allergic skin reaction	Buchanan et al. 2007^a^

FLB, *Fructolactobacilli*; SPT, skin prick test.

TNF-α, MCP-1, IL6: inflammation markers released after cell line challenge with ATI isolate.

^a^ Total albumin/globulin extract.

^b^ Identification of ATI based on molecular weight in SDS-PAGE.

### 3.1. Effect of yeast and *Fructolactobacilli* fermentation on ATIs

In several publications, the impact of yeast or *Fructolactobacilli* fermentation has been studied. Only the impact of fermentation will be discussed in this section. The impact of fermentation and baking will be discussed in the succeeding section.

Sourdough fermentation is known to have proteolytic activity resulting in digestion of wheat proteins including ATIs (39, 46, 48, 51, 57). During the fermentation process, specific sourdough FLB were found to hydrolyze salt-soluble proteins including ATIs (e.g., *Lactobacillus alimentarius 15M, Lactobacillus brevis 14G, Lactobacillus sanfranciscensis 7A,* and *Lactobacillus hilgardii 51B*), while other FLB did not exhibit this hydrolytic activity (46, 48).

The overview from Table 2 shows that the total quantity of extracted ATI can increase (45), remain equal, or decrease (40), depending on the type of FLB, type of extraction, and type of measurement employed. For yeast fermentation, the quantity remains equal (45). Binding of IgE from patients with wheat allergies to the total ATI extract was almost completely absent after FLB fermentation with two selected strains (44). No difference was found in the total extracted ATI level in a direct comparison of yeast vs. FLB fermentation (50). When various studies are compared, it is evident that there is a lack of consistent findings regarding the quantity and IgE binding properties of ATIs in relation to FLB and yeast fermentation.

When ATIs were partially extracted with tris-HCL, NaCl, or chloroform–methanol, generally the amounts of ATI were substantially reduced by FLB fermentation (22, 45, 48, 49), although no decrease was observed with some FLB (48). For yeast fermentation, the amount of ATI in the partial extracts remained equal (45).

Won et al. (50) found no change in abundance of ammonium bicarbonate-extractable CM3 after FLB and yeast fermentation. Also, no difference with a straight yeast dough was observed. The absence of IgE binding from patients with wheat allergies was observed in the NaCl extract after FLB fermentation (47). This conflicts with the findings of Stefańska et al. (44), where binding still remained in the total extract. Amylase inhibition from dough, assayed in a NaCl extract, decreased by 22% and 29% with FLB fermentation (46), indicating that it was not completely lost.

In cell line studies, ATI extracts decreased the inflammation markers TNF-α and monocyte chemoattractant protein-1 release after FLB fermentation when compared with yeast fermentation (46). In the only *in vivo* animal study encountered, important markers for inflammation TNF-α and IL6 were studied in mice. When mice were fed with isolated ATIs in combination with high ATI degrading FLB, there was a 55% decrease in TNF-α release from splenocytes in serum and a 50% decrease in the increase of IL6 compared with feeding them with low ATI degrading FLB or without FLB (40).

Understanding the mechanisms of action of bacteria during sourdough fermentation could help develop food processing methods to mitigate ATI pathology. Especially the specific proteolysis of ATIs by specific species of FLB are of interest.

### 3.2. Effect of fermentation and heating during baking on ATIs

Heat treatment is one of the most common food processing methods. Next to potential conformational changes in proteins, Maillard reaction products (MRP) are readily formed. The Maillard reaction modulates the allergenicity of proteins in numerous ways by enhancing (increased uptake, delayed digestion, new epitopes) or reducing allergenicity (reduced uptake of larger polymers, modulating epitopes; 26).

After baking of FLB and yeast-fermented doughs, the amount of ATIs in the total extract is reduced (47, 50), and no differences were found between FLB and yeast fermentations after baking (50). Binding of IgE from patients with wheat allergies with ATIs was absent in FLB-fermented breads and reduced (47) or absent in the crust and the crumb in yeast-fermented breads (56). Surprisingly, the binding of IgE from patients with wheat allergies returned when yeast-fermented bread was hydrolyzed with pancreatin (56). Although aggregation of ATIs into protein carbohydrate complexes during baking cannot completely be ruled out, the authors conclude that heat treatment and subsequent proteolytic digestion eliminates the allergenicity of the ATIs (56). In a partial extract from FLB and yeast-fermented breads, the ATI quantities were equally reduced. In FLB-fermented breads, more monomers and more di/tetrameric ATIs were present (51), indicating that the process of fermentation and baking can impact the quaternary structure of ATIs. The CM3 extractability was equally reduced after baking of FLB and yeast-fermented doughs (50).

Sodium acetate-extracted proteins from various types of bread were analysed for their trypsin and chymotrypsin inhibitory (TI and CI, respectively) effects. Trypsin (TI)/chymotrypsin (CI) inhibition for wheat, rye, and mixed cereals was the highest for bread, followed by flour, and dough had the lowest inhibitory effect. Wholemeal showed no TI. TI of rye breads were the highest followed by wheat/mixed cereals. CI of wheat was larger than rye, followed by wholemeal and then mixed cereals (52). Heat treatment of bread dough seemed to decrease the free ATI level slightly in the crumb, but in the crust, ATI could not be detected anymore, probably due to heavy cross-linking of proteins and abundant Maillard reaction products. Pepsin followed by pancreatic *in vitro* digestion showed that the ATIs in yeast dough were not degraded to a large extent, but in the bread crumb, they quickly disappeared, although some ATI isoforms were resistant to degradation. This was confirmed with ATI polyclonal antibody immunoblotting (37).

During the baking process, most of the ATI enzymatic inhibitory activity is lost except for ATIs present in the decoration of bread, e.g., dusting flour (52). This is likely due to the low water activity in the dusting flour, which protects the proteins from denaturation. The loss in enzymatic inhibitory activity does not imply that the allergenicity of ATIs is lost as well, and there is evidence in other cereal processes, e.g., brewing, that allergenicity remains or is even potentiated. Similar inhibitors from barley (BASI) have been investigated in detail during the brewing process. There is quite extensive molecular information on the food processing of ATI and other pathogenesis-related proteins during beer brewing that is excellently reviewed (a.o., glycation, acylation, disulphide bond reduction, denaturation/conformational changes, etc.) (58).

In addition to proteolytic modification and denaturation, glycation of proteins may also occur during food processing. For barley processing into beer, it was speculated that the large difference in wort pH and pI of the protein may lead to its unfolding, making the lysine residues available for glycation (59, 60). Complete unfolding was only possible when all disulphide bonds were reduced (59). The glycation of the lipid transfer protein (LTP) is important for the final foaming properties of beer (60). Barley contains LTP and barley amylase subtilisin inhibitor (BASI), which are similar to ATIs from wheat. There is evidence that the glycated BASI is more allergenic than the non-glycated versions (27, 61, 62). Thus, the allergenicity of epitopes can be mitigated during heating, but epitopes can also become exposed, generated, or even potentiated during the food processing. Glycated CM16 isolated from *Triticum durum* flour has a strongly increased binding to IgE of serum from patients with baker's asthma substantially, when compared with non-glycated CM16 (27). It is not clear how the CM16 became glycated. Glycation of ATIs during food processing has not been investigated.

Publications on the effect of heating on enzyme inhibition are conflicting. Naji et al. (63) reported an increase in enzyme inhibition in white bread after baking. Others found a decrease during baking of bread and boiling of pasta (55, 64). The remaining activity was either absent (in sugar-rich baked goods or several white bread types 55) or decreased substantially to approximately 12.5% in the central part of the bread or to approximately 7% in the peripheral part (63). Gélinas et al. (55) found generally a lower inhibitory activity of bread crust than in the crumb, but in some bread, the reverse was found. Dusting flour was the main contributor to the inhibitory activity of the crust. They also demonstrated that the presence of sugar interfered with the inhibitory activity (55), and it is likely that, next to ATIs, sugar inhibited amylase via reaction product inhibition. As the authors did not state that they removed the sugar in the extract for the enzyme assay, this could explain why no inhibitory activity was found in sugar-rich products. In the crumb of wholemeal bread, the inhibitory activity was among the highest of commercial breads (55) or it was absent (64).

Gluten, which is a common ingredient derived from wheat and used to increase the baking quality of wheat, has even higher enzyme inhibitory activity than wheat flour, similar to the potent amylase-inhibitor medicine acarbose (55). Finally, it is likely that the mitigation of enzyme inhibitory activities of ATI during bread processing does not result automatically in mitigation of its allergic properties.

### 3.3. Effect of heat treatment during drying and boiling on ATIs

The type of process has also a major effect on the level of allergenic low molecular weight proteins such as ATI: barley malting (germination, kilning, no heat) decreased the water extractable LTP to 36% of its original content, whereas water extractable CM16 increased by 8%. After cous-cous preparation from *T. durum* (forming, cooking, drying), only 4%–26% of the ATIs were water extractable (65).

When pasta samples that were dried under mild or harsh conditions were subjected to *in vitro* digestion, the resulting digests from the albumin/globulin fraction exhibited comparable reactivity in competitive ELISA with IgE from sera of wheat-allergic patients. Drying at temperatures > 110°C seemed to increase the *in vitro* IgE binding (66). This could not be confirmed in another study with pastas dried at different temperatures. No IgE binding to the low molecular weight protein fraction could be observed in dried, cooked, and *in vitro* pepsin/pancreatin digested pasta (53). It can be speculated that in this study, the cooking procedure was different, resulting in cooking loss of ATI or that the degradation of the proteins was so fast that the peptides disappeared from the gel before immunoblotting. Drying of pasta for 4 min at 150°C did reduce the inhibitory activity to a level similar to that of flour (67).

ATIs from *T. durum* semolina were able to react with the serum from wheat allergy-specific IgE positive patients with gastrointestinal problems upon wheat ingestion, but not with the serum from wheat allergy-specific IgE negative patients with wheat-based gastrointestinal problems. Upon pasta processing (high shear and drying at elevated temperature) and subsequent cooking, no IgE binding could be observed anymore, indicating a loss of epitope recognition, as the ATIs were still detected by Coomassie staining in the raw and cooked pasta (38). Pepsin digestion was able to reduce the IgE binding of ATI with serum from wheat allergy-specific IgE positive patients (38). Unfortunately, the immunoblots did not show the full spectrum of the proteins and peptides, and there is no certainty that the degraded peptides were still recognized by the IgE, or that the peptides were retained on the immunoblot during overnight incubation with sera. A similar study was performed by Mamone et al. (54). It was concluded that ATIs (CM 2, 3, and 16) and a large portion of nsLTP appeared in the cooking water, as they could not detect ATIs in the salt extract from cooked pasta but only in the cooking water. The authors provide conflicting results as in the same publication, ATIs could be extracted from cooked pasta. However, upon *in vitro* digestion, the ATIs from the raw pasta survived digestion, but those from the cooked pasta did not. nsLTP were resistant to hydrolysis in the raw and in the uncooked pasta (54). Simonato et al. (38) showed that ATIs underwent modifications during processing, still being present in cooked pasta, but losing their IgE binding capacity. The discrepancy among the studies remains unexplained, except that it can be observed in the *in vitro* gastric or gastric and duodenal digestion (54). Furthermore, in the undigested pasta (*t* = 0), no other wheat proteins were present, indicating that pepsin probably remained active in the lithium dodecyl sulphate extraction medium (68) prior to the electrophoresis. The loss in IgE binding capacity can therefore be the result of ongoing proteolysis prior to the Western blotting.

In ELISA testing, binding of IgE from wheat-allergic patients to salt extracts from flour, bread, and pasta decreased when the product had been heated (pasta, bread) and even further when toasted (34). The ELISA inhibition assay with the salt extract from raw flour showed that the salt extracts from the processed food still showed large inhibitory effects (47%–89%), indicating that the epitopes were still intact (34). As the protein yields of the extracts were not reported, it is not clear whether these effects are due to a decrease in extractability or due to the loss of allergenic epitopes. Allergenic 20 kDa (globulin-like) and 32 kDa proteins were identified in untoasted white and wholemeal bread and in cooked fresh and in dried pasta (34). Next to this, some ATI reactivity (approximately 15 kDa) can be observed qualitatively in the Western blots from the extracts of French bread, pizza, and white and wholemeal tin bread; moderate activity was seen in the Western blots from extracts of biscuits; and strong ATI reactivity can be observed in unboiled white and wholemeal pasta and in fresh white pasta. This finding indicates that either heating pasta or toasting bread can lead to the elimination of allergenic epitopes or a decrease in the protein extractability.

In skin prick tests, the salt extractable proteins from flour and wholemeal tin bread gave an equally large allergic reaction with cereal allergic patients. For toasted bread and cooked pasta, the reaction was significantly less [wheal sizes of 79% and 64% less, respectively (34)].

## 4. Discussion and conclusions

**Figure 1:**
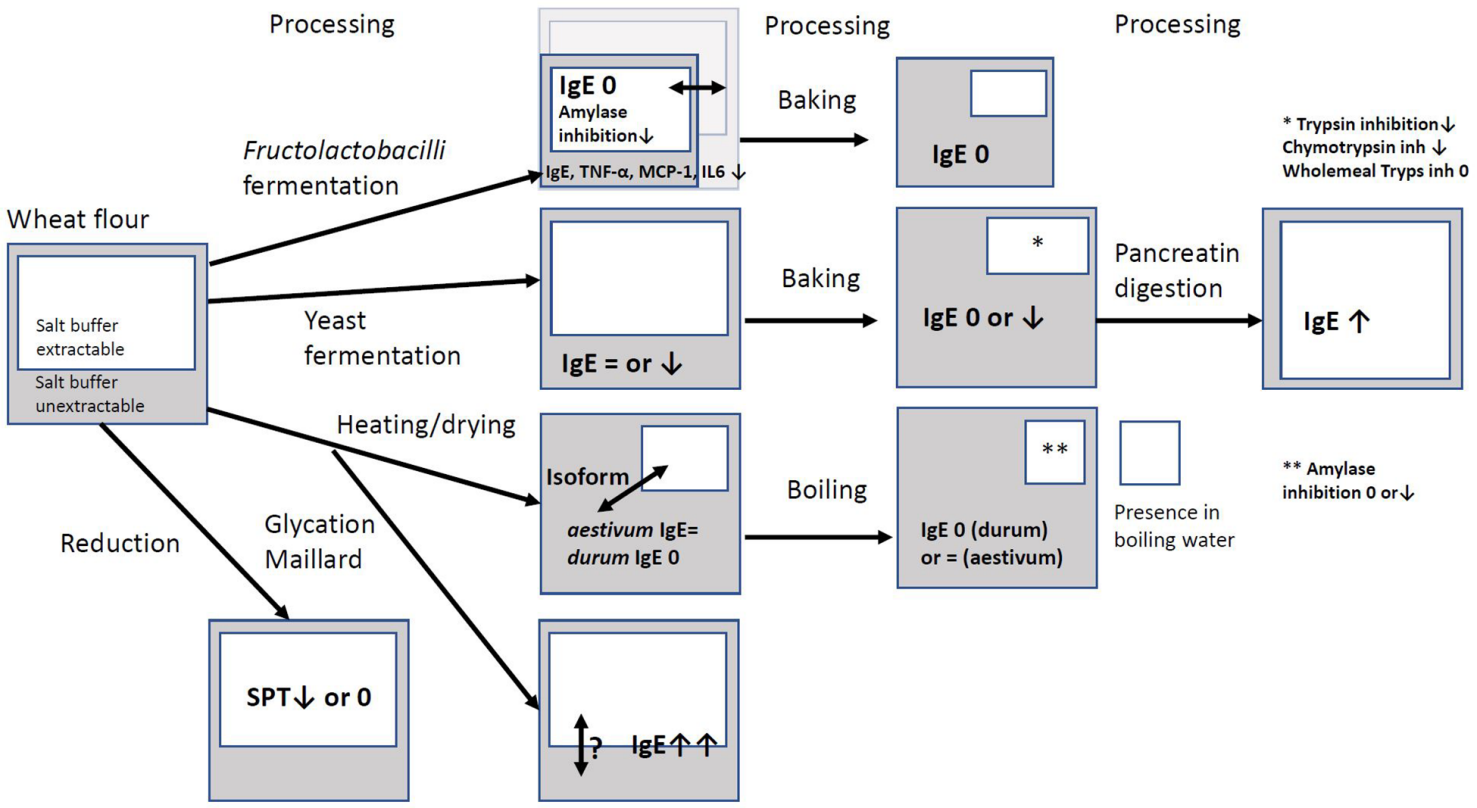
Overview of impact of processing on extractability and amount of ATI and binding to IgE from wheat-allergic patients, skin prick test, enzyme inhibition, or cell line studies. White box: amount of ATI extractable in salt buffer; Grey box: amount of ATI unextractable in salt buffer; remark in the box: biofunctionality of partial extract (white) or total extract (grey), no remark means no study encountered; biofunctionality: ↑ increase, ↓ decrease, = equal, 0 absent IgE: binding of IgE from wheat-allergic patients to ATI; SPT = skin prick test; TNF-α, MCP-1, IL6: inflammation markers released after cell line challenge with ATI isolate; ↔ larger or smaller amount depending on the study.

The potential pathogenic effects of ATIs in NCWS and their allergenic properties in individuals with baker's allergies and wheat allergies have been well-documented. Therefore, the exploration of strategies to mitigate these negative biofunctionalities through food processing is of interest. To date, there is a lack of *in vivo* studies involving human subjects that have reported the utilization of ATIs that were modified by food processing. Early studies with modification of the albumin globulin fractions with thioredoxin/thioreductase NADPH showed that altering the redox status of this fraction can reduce the allergenic effects on the skin with dogs (31) and the skin wheal reaction of one child with baker's asthma (29). Isolated ATIs that were fed in combination with highly degrading FLB to mice showed a reduction in intestinal inflammation markers. The results of this review can be summarised in Figure 1. When applying FLB fermentation with various FLB, the impact on ATIs is not always clear. Only a few studies used the total extract of baked bread or boiled pasta. With FLB-fermented bread, but also yeast-fermented bread, binding of IgE from wheat-allergic patients to ATIs could be decreased or even absent. With FLB-fermented bread, this varied widely depending on the FLB used. It was observed that some ATIs were resistant to proteolysis after baking, since IgE binding to ATIs remained. Furthermore, heating dough could decrease the extractability of isoform 0.19 by 40%, but also substantial increases in extractability by 39% to more than 300% of 0.19, dimeric, CM3, and CM16 were observed (13). This indicates that isoforms can be differently affected by heating. Also, the dual impact on the 0.19 inhibitor is interesting, as it is possible that variations in the influence of food processing may occur within one isoform. This warrants further comprehensive investigations. In addition, it is crucial to explore the effects of other modifications, such as glycosylation, formation of Maillard intermediate products, and (partial) reduction on the pathogenicity of ATIs.

The studies of the impact of food processing on ATI pathology is hindered by the anomalous solubility behaviour of various ATI isoforms and the impact which processing itself has on the extractability. The use of chaotropic agents in combination with reducing agents are required to extract the maximum amount ATIs from processed food. A quantitative extraction from processed food is difficult to achieve. Such extraction will alter its conformation and secondary structure, which in turn may alter their pathogenic effects, bioactivity, and immunoreactivity. This also urges for further research. Finally, the available literature on *in vitro* pathogenic effects (IgE binding, cell line studies) is currently limited, and more studies are required to conclude on a broader spectrum of *in vitro* pathogenic effects of ATIs. Finally, *in vivo* studies—especially in humans—are required to establish the impact of processing on the pathogenic properties of ATIs.

## References

[1] Weegels PL. The future of bread in view of its contribution to nutrient intake as a starchy staple food. Plant Foods Hum Nutr. (2019) 74(1):1–9. 10.1007/s11130-019-0713-6 30637605

[2] Flacco ME Manzoli L De Giorgio R Gasbarrini A Cicchetti A Bravi F Costs of irritable bowel syndrome in European countries with universal healthcare coverage: a meta-analysis. Eur Rev Med Pharmacol Sci. (2019) 23(7):2986–3000. 10.26355/eurrev_201904_17580 31002149

[3] Huebener S Tanaka CK Uhde M Zone JJ Vensel WH Kasarda DD Specific nongluten proteins of wheat are novel target antigens in celiac disease humoral response. J Proteome Res. (2015) 14:503–11. 10.1021/pr500809b 25329597 PMC4285749

[4] Junker Y Zeissig S Kim SJ Barisani D Wieser H Leffler DA Wheat amylase trypsin inhibitors drive intestinal inflammation via activation of toll-like receptor 4. J Exp Med. (2012) 209(13):2395–408. 10.1084/jem.20102660 23209313 PMC3526354

[5] Caminero A Galipeau HJ McCarville JL Johnston CW Bernier SP Russell AK Duodenal bacteria from patients with celiac disease and healthy subjects distinctly affect gluten breakdown and immunogenicity. Gastroenterology. (2016) 151(4):670–83. 10.1053/j.gastro.2016.06.041 27373514

[6] Gao H Jorgensen R Raghunath R Nagisetty S Ng PK Gangur V. Creating hypo-/nonallergenic wheat products using processing methods: fact or fiction? Comp Rev Food Sci Food Safety. (2021) 20(6):6089–115. 10.1111/1541-4337.12830 34455695

[7] Prieto JA Kelfkens M Weegels PL Hamer RJ. Variations in the Gliadin pattern of flour and isolated gluten on nitrogen application. Z Lebensm Unters Forsch. (1992) 194:337–43. 10.1007/BF01193216

[8] Weegels PL Hamer RJ Schofield JD. Functional properties of wheat glutenin. J Cereal Sci. (1996) 23(1):1–17. 10.1006/jcrs.1996.0001

[9] Osborne TB. *The proteins of the wheat kerne!.* Pub!. 84. Carnegie Inst., Washington, D.C. (1907).

[10] Wasik RJ Bushuk W. Studies of glutenin. V. Note on additional preparative methods. Cereal Chem. (1974) 51:112–8.

[11] Prieto JA Weegels PL Hamer RJ. Functional properties of low Mr wheat proteins. I. Isolation, characterization and comparison with other reported low Mr wheat proteins. J Cereal Sci. (1993) 17(3):203–20. 10.1006/jcrs.1993.1020

[12] Weegels PL Hamer RJ Schofield JD. RP–HPLC and capillary electrophoresis of subunits from glutenin isolated by SDS and Osborne fractionation. J Cereal Sci. (1995) 22(3):211–24. 10.1006/jcrs.1995.0058

[13] Wang X Appels R Zhang X Bekes F Diepeveen D Ma W Solubility variation of wheat dough proteins: a practical way to track protein behaviors in dough processing. Food Chem. (2020) 312:126038. 10.1016/j.foodchem.2019.126038 31896458

[14] Simonetti E Bosi S Negri L Baffoni L Masoni A Marotti I Molecular phylogenetic analysis of amylase trypsin inhibitors (ATIs) from a selection of ancient and modern wheat. J Cereal Sci. (2022) 103441. 10.1016/j.jcs.2022.103441

[15] Baatjies R Meijster T Heederik D Jeebhay MF. Exposure–response relationships for inhalant wheat allergen exposure and asthma. Occup Environ Med. (2015) 72(3):200–7. 10.1136/oemed-2013-101853 25535033

[16] Wang JR Zhang L Wei YM Yan ZH Baum BR Nevo E Sequence polymorphisms and relationships of dimeric *α*-amylase inhibitor genes in the B genomes of *Triticum* and S genomes of *Aegilops*. Plant Sci. (2007) 173(1):1–11. 10.1016/j.plantsci.2007.03.006

[17] Sander I Rozynek P Rihs HP Van Kampen V Chew FT Lee WS Multiple wheat flour allergens and cross-reactive carbohydrate determinants bind IgE in baker’s asthma. Allergy. (2011) 66(9):1208–15. 10.1111/j.1398-9995.2011.02636.x 21557753

[18] Geisslitz S Shewry P Brouns F Schuppan D America AHP Caio GPI Wheat ATIs: characteristics and role in human disease. Front Nutr. (2021) 8:667370. 10.3389/fnut.2021.667370 34124122 PMC8192694

[19] Lupi R Denery-Papini S Claude M Tranquet O Drouet M Masci S Thermal treatment reduces gliadin recognition by IgE, but a subsequent digestion and epithelial crossing permits recovery. Food Res Int. (2019) 118:22–31. 10.1016/j.foodres.2018.02.011 30898348

[20] García-Olmedo F García-Faure R. A new method for the estimation of common wheat (*T. aestivum* L.) in pasta products. Lebensm Wiss Technol. (1969) 2:94–6.

[21] Weegels PL. Depolymerisation and re-polymerisation of wheat glutenin during dough processing and effects of low Mr wheat proteins. PhD thesis UK: University of London (1994).

[22] Boakye PG Kougblenou I Murai T Okyere AY Anderson J Bajgain P Impact of sourdough fermentation on FODMAPs and amylase-trypsin inhibitor levels in wheat dough. J Cereal Sci. (2022) 108:103574. 10.1016/j.jcs.2022.103574

[23] Geisslitz S Longin CFH Koehler P Scherf KA. Comparative quantitative LC–MS/MS analysis of 13 amylase/trypsin inhibitors in ancient and modern *Triticum* species. Nat Sci Rep. (2020) 10(1):14570. 10.1038/s41598-020-71413-z PMC7471314 32883982

[24] Wong JH Cai N Tanaka CK Vensel WH Hurkman WJ Buchanan BB. Thioredoxin reduction alters the solubility of proteins of wheat starchy endosperm: an early event in cereal germination. Plant Cell Physiol. (2004) 45(4):407–15. 10.1093/pcp/pch044 15111715

[25] Lagrain B De Vleeschouwer K Rombouts I Brijs K Hendrickx ME Delcour JA. The kinetics of *β*-elimination of cystine and the formation of lanthionine in gliadin. J Agri Food Chem. (2010) 58(19):10761–7. 10.1021/jf102575r 20836554

[26] Teodorowicz M Van Neerven J Savelkoul H. Food processing: the influence of the Maillard reaction on immunogenicity and allergenicity of food proteins. Nutrients. (2017) 9(8):835. 10.3390/nu9080835 28777346 PMC5579628

[27] Sanchez-Monge R Gomez L Barber D Lopez-Otin C Armentia A Salcedo G. Wheat and barley allergens associated with baker’s asthma. Glycosylated subunits of the alpha-amylase-inhibitor family have enhanced IgE-binding capacity. Biochem J. (1992) 281(Pt 2):401. 10.1042/bj2810401 1736890 PMC1130698

[28] Weegels PL Flissebaalje TH Hamer RJ. Factors affecting the extractability of the glutenin macropolymer. Cereal Chem. (1995) 71(3):308–9.

[29] Matsumoto T Shimada Y Hirai S. Mitigated binding of IgE to thioredoxin-treated salt-soluble wheat allergens in a child with baker’s asthma. Ann Allergy Asthma Immunol. (2007) 98(6):599–600. 10.1016/S1081-1206(10)60746-6 17605187

[30] Jensen JM Hägglund P Christensen HEM Svensson B. Inactivation of barley limit dextrinase inhibitor by thioredoxin-catalysed disulfide reduction. FEBS Lett. (2012) 586(16):2479–82. 10.1016/j.febslet.2012.06.009 22728244

[31] Buchanan BB Adamidi C Lozano RM Yee BC Momma M Kobrehel K Thioredoxin-linked mitigation of allergic responses to wheat. Proc Nation Acad Sci. (1997) 94(10):5372–7. 10.1073/pnas.94.10.5372 PMC24685 9144244

[32] Mosolov VV Shul’gin MN. Protein inhibitors of microbial proteinases from wheat, rye and triticale. Planta. (1986) 167(4):595–600. 10.1007/BF00391238 24240378

[33] Oneda H Lee S Inouye K. Inhibitory effect of 0.19 *α*-amylase inhibitor from wheat kernel on the activity of porcine pancreas *α*-amylase and its thermal stability. J Biochem. (2004) 135(3):421–7. 10.1093/jb/mvh050 15113841

[34] De Gregorio M Armentia A Díaz-Perales A Palacin A Dueñas-Laita A Martín B Salt-soluble proteins from wheat-derived foodstuffs show lower allergenic potency than those from raw flour. J Agric Food Chem. (2009) 57(8):3325–30. 10.1021/jf803475v 19275238

[35] Simonato B De Lazzari F Pasini G Polato F Giannattasio M Gemignani C IgE binding to soluble and insoluble wheat flour proteins in atopic and non-atopic patients suffering from gastrointestinal symptoms after wheat ingestion. Clin Exp Allergy. (2001) 31(11):1771–8. 10.1046/j.1365-2222.2001.01200.x 11696054

[36] O’Connor CM McGeeney KF. Isolation and characterization of four inhibitors from wheat flour which display differential inhibition specificities for human salivary and human pancreatic *α*-amylases. Biochim Biophys Acta Enzymol. (1981) 658(2):387–96. 10.1016/0005-2744(81)90309-0 6166323

[37] Pasini G Simonato B Giannattasio M Peruffo AD Curioni A. Modifications of wheat flour proteins during in vitro digestion of bread dough, crumb, and crust: an electrophoretic and immunological study. J Agric Food Chem. (2001) 49(5):2254–61. 10.1021/jf0014260 11368585

[38] Simonato B Pasini G De Zorzi M Vegro M Curioni A. Potential allergens in durum wheat semolina and pasta: fat during cooking and digestion. Ital J Food Sci. (2004) 16(2):151–64.

[39] Di Cagno R De Angelis M Alfonsi G de Vincenzi M Silano M Vincentians O Pasta made from durum wheat semolina fermented with selected lactobacilli as a tool for a potential decrease of the gluten intolerance. J. Agric Food Chem. (2005) 53(11):4393–402. 10.1021/jf048341 15913301

[40] Caminero A McCarville JL Zevallos VF Pigrau M Xuechen BY Jury J Lactobacilli degrade wheat amylase trypsin inhibitors to reduce intestinal dysfunction induced by immunogenic wheat proteins. Gastroenterology. (2019) 156(8):2266–80. 10.1053/j.gastro.2019.02.028 30802444

[41] Islamov RA Fursov OV. Bifunctional inhibitor of [alpha]-amylase/trypsin from wheat grain. Appl Biochem Microbiol. (2007) 43(4):379. 10.1134/S0003683807040035 17929568

[42] Gvozdeva EL Valueva TA Mosolov VV. Enzymatic oxidation of the bifunctional wheat inhibitor of subtilisin and endogenous *α*-amylase. FEBS Lett. (1993) 334(1):72–4. 10.1016/0014-5793(93)81683-Q 8224231

[43] Kobrehel K Yee BC Buchanan BB. Role of the NADP/thioredoxin system in the reduction of alpha-amylase and trypsin inhibitor proteins. J Biol Chem. (1991) 266(24):16135–40. 10.1016/S0021-9258(18)98526-1 1874751

[44] Stefańska I Piasecka-Jóźwiak K Kotyrba D Kolenda M Stecka KM. Selection of lactic acid bacteria strains for the hydrolysis of allergenic proteins of wheat flour. J Sci Food Agric. (2016) 96(11):3897–905. 10.1002/jsfa.7588 26693837

[45] Yin Y Wang J Yang S Feng J Jia F Zhang C. Protein degradation in wheat sourdough fermentation with *Lactobacillus plantarum* M616. Interdisciplinary sciences: comp. Life Sci. (2015) 7:205–10. 10.1007/s12539-015-0262-0 26199213

[46] Huang X Schuppan D Rojas Tovar LE Zevallos VF Loponen J Gänzle M. Sourdough fermentation degrades wheat alpha-amylase/trypsin inhibitor (ATI) and reduces pro-inflammatory activity. Foods. (2020) 9(7):943. 10.3390/foods9070943 32708800 PMC7404469

[47] De Angelis M Rizzello CG Scala E De Simone C Farris GA Turrini F Probiotic preparation has the capacity to hydrolyze proteins responsible for wheat allergy. J. Food Prot. (2007) 70(1):135–44. 10.4315/0362-028X-70.1.135 17265872

[48] Di Cagno R De Angelis M Lavermicocca P De Vincenzi M Giovannini C Faccia M Proteolysis by sourdough lactic acid bacteria: effects on wheat flour protein fractions and gliadin peptides involved in human cereal intolerance. Appl Environ Microbiol. (2002) 68:623–33. 10.1128/aem.68.2.623-633.2002 11823200 PMC126681

[49] Fraberger V Ladurner M Nemec A Grunwald-Gruber C Call LM Hochegger R Insights into the potential of sourdough-related lactic acid bacteria to degrade proteins in wheat. Microorganisms. (2020) 8(11):1689. 10.3390/microorganisms8111689 33143014 PMC7693696

[50] Won S Curtis J Gänzle M. LC-MS/MS quantitation of *α*-amylase/trypsin inhibitor CM3 and glutathione during wheat sourdough breadmaking. J Appl Microbiol. (2022) 133(1):120–9. 10.1111/jam.15346 34724302

[51] Laatikainen R Koskenpato J Hongisto SM Loponen J Poussa T Huang X Pilot study: comparison of sourdough wheat bread and yeast-fermented wheat bread in individuals with wheat sensitivity and irritable bowel syndrome. Nutrients. (2017) 9:1215. 10.3390/nu9111215 29113045 PMC5707687

[52] Kostekli M Karakaya S. Protease inhibitors in various flours and breads: effect of fermentation, baking and in vitro digestion on trypsin and chymotrypsin inhibitory activities. Food Chem. (2017) 224:62–8. 10.1016/j.foodchem.2016.12.048 28159294

[53] De Zorzi M Curioni A Simonato B Giannattasio M Pasini G. Effect of pasta drying temperature on gastrointestinal digestibility and allergenicity of durum wheat proteins. Food Chem. (2007) 104(1):353–63. 10.1016/j.foodchem.2006.11.057

[54] Mamone G Nitride C Picariello G Addeo F Ferranti P Mackie A. Tracking the fate of pasta (*T. durum semolina*) immunogenic proteins by in vitro simulated digestion. J Agric Food Chem. (2015) 63(10):2660–7. 10.1021/jf505461x 25682706

[55] Gélinas P McKinnon C Gagnon F. Inhibitory activity towards human *α*-amylase in cereal foods. LWT. (2018) 93:268–73. 10.1016/j.lwt.2018.03.049

[56] Simonato B Pasini G Giannattasio M Peruffo AD De Lazzari F Curioni A. Food allergy to wheat products: the effect of bread baking and in vitro digestion on wheat allergenic proteins. A study with bread dough, crumb, and crust. J Agric Food Chem. (2001) 49(11):5668–73. 10.1021/jf0104984 11714375

[57] Zevallos VF Raker V Tenzer S Jimenez-Calvente C Ashfaq-Khan M Rüssel N Nutritional wheat amylase-trypsin inhibitors promote intestinal inflammation via activation of myeloid cells. Gastroenterology. (2017) 152(5):1100–13. 10.1053/j.gastro.2016.12.006 27993525

[58] Stanislava G. A review: the role of barley seed pathogenesis-related proteins (PRs) in beer production. J Instit Brew. (2010) 116(2):111–24. 10.1002/j.2050-0416.2010.tb00407.x

[59] Jégou S Douliez JP Mollé D Boivin P Marion D. Purification and structural characterization of LTP1 polypeptides from beer. J Agric Food Chem. (2000) 48(10):5023–9. 10.1021/jf000075m 11052772

[60] Jégou S Douliez JP Mollé D Boivin P Marion D. Evidence of the glycation and denaturation of LTP1 during the malting and brewing process. J Agric Food Chem. (2001) 49(10):4942–9. 10.1021/jf010487a 11600048

[61] Garcia-Casado G Sanchez-Monge R Chrispeels MJ Armentia A Salcedo G Gomez L. Role of complex asparagine-linked glycans in the allergenicity of plant glycoproteins. Glycobiology. (1996) 6(4):471–7. 10.1093/glycob/6.4.471 8842712

[62] Mena M Sanchez-Monge R Gomez L Salcedo G Carbonero P. A major barley allergen associated with baker’s asthma disease is a glycosylated monomeric inhibitor of insect *α*-amylase: cDNA cloning and chromosomal location of the gene. Plant Mol Biol. (1992) 20(3):451–8. 10.1007/BF00040604 1421148

[63] Naji EZ Abood SC Iqdiam BM. A study of the bread preparation stages on salivary alpha-amylase inhibitors extracted from wheat variety IPA 99. J Tokrit Univ Agric Sci. (2011) 11:1–7.

[64] Granum PE. Studies on *α*-amylase inhibitors in foods. Food Chem. (1979) 4(3):173–8. 10.1016/0308-8146(79)90001-3

[65] Flodrová D Benkovská D Laštovičková M. Study of quantitative changes of cereal allergenic proteins after food processing. J Sci Food Agric. (2015) 95(5):983–90. 10.1002/jsfa.6773 24912629

[66] Petitot M Brossard C Barron C Larré C Morel MH Micard V. Modification of pasta structure induced by high drying temperatures. Effects on the in vitro digestibility of protein and starch fractions and the potential allergenicity of protein hydrolysates. Food Chem. (2009) 116(2):401–12. 10.1016/j.foodchem.2009.01.001

[67] Gélinas P Gagnon F. Inhibitory activity towards human *α*-amylase in wheat flour and gluten. Int J Food Sci Technol. (2018) 53(2):467–74. 10.1111/ijfs.13605

[68] Guzman ML Marques MR Me MEO Stippler ES. Enzymatic activity in the presence of surfactants commonly used in dissolution media, part 1: pepsin. Res Pharma Sci. (2016) 6:15–9. 10.1016/j.rinphs.2016.02.002 PMC4796717 27047734

